# Chinese Herb and Formulas for Promoting Blood Circulation and Removing Blood Stasis and Antiplatelet Therapies

**DOI:** 10.1155/2012/184503

**Published:** 2012-02-21

**Authors:** Yue Liu, Hui-Jun Yin, Da-zhuo Shi, Ke-ji Chen

**Affiliations:** Cardiovascular Diseases Center, Xiyuan Hospital of China Academy of Chinese Medical Sciences, Beijing 100091, China

## Abstract

Atherothrombosis, which directly threatens people's health and lives, is the main cause of morbidity and mortality all over the world. Platelets play a key role in the development of acute coronary syndromes (ACSs) and contribute to cardiovascular events. Oral antiplatelet drugs are a milestone in the therapy of cardiovascular atherothrombotic diseases. In recent years, many reports have shown the possibility that “resistance” to oral anti-platelet drugs and many adverse reactions, such as serious bleeding risk, which provides an impetus for developing new anti-platelet drugs possesses highly efficiency and fewer adverse effects. Study on the blood stasis syndrome and promoting blood circulation and removing blood stasis is the most active field of research of integration of traditional and western medicine in China. Blood-stasis syndrome and platelet activation have close relationship, many Chinese herb and formulas for promoting blood circulation and removing blood stasis possess definite anti-platelet effect. This paper covers the progress of anti-platelet mechanism of Chinese herb and formulas for promoting blood circulation and removing blood stasis and is to be deeply discussed in further research.

## 1. Introduction

Cardiovascular and cerebrovascular events have become the major killer of people's health and life all over the world. Rupture of atherosclerotic plaque in an artery wall and the ensuing thrombotic events are the triggers for acute ischemic injury. Activated platelets play a pivotal role in the formation of pathogenic thrombi underlying acute clinical manifestations of vascular atherothrombotic disease. Oral antiplatelet drugs are a milestone in the therapy of cardiovascular atherothrombotic diseases and provide the primary and secondary prevention strategy to combat these diseases. Efficient antiplatelet therapy can make the death rates of heart disease and stroke decline by about 25% [1, 2]. Commonly used oral antiplatelet drugs include cyclooxygenase inhibitor aspirin, the glycoprotein IIb/IIIa inhibitor ReoPro, and the P_2_Y_12_ inhibitor clopidogrel, et al. Many clinical studies show that dual antiplatelet therapy with aspirin and clopidogrel is currently the standard of drugs for prevention of adverse cardiovascular events in most patients at high risk owing to acute coronary syndromes or recent placement of a stent.

But along with prolonging of treatment by dual or triple antiplatelet drugs, the effectiveness and security have garnered particular attention in clinic. Despite their proven benefit, recurrent cardiovascular events still occur in those taking antiplatelet drugs. This has led to the concept of antiplatelet resistance [3], most commonly aspirin resistance as this drug is the cornerstone of most regimens. Although there are some debates on definition and mechanism of antiplatelet resistance [4, 5], it cannot be denied that it has important clinical significance. At the same time, numerous adverse reactions including serious bleeding risk (digestive and nervous systems) and combination with PPIs and statin [6, 7], which limit the clinical practice of antiplatelet drugs. So developed novel classes of antiplatelet agents possess high efficiency, and fewer adverse effects have been always the research focus for prevention of cardiovascular disease. Modern medicine and pharmacology has done a lot of valuable exploration, newer agents are in development recent years that include prasugrel, cangrelor, ticagrelor, and vorapaxar, et al. [8].

Study on the *blood stasis syndrome* (BSS) and *promoting blood circulation and removing blood stasis* (PBCRBS) is the most active field of research of integration of traditional and western medicine in China. During the past 50 years, much significant progress has been made from theory, experiments to clinic fields based on the inherit, and innovation of thoughts in traditional Chinese medicine [9], to clarify the treatment regulations and principles of PBCRBS, which has already got consensus in medical community in China. A lot of formulas for PBCRBS (see Table 1) have showed great antiplatelet effect in clinic, and most of them are the Chinese patent drugs. On the prevention of atherosclerosis or vulnerable plaque, Chinese and Western medicine have the consensus that stabling plaque and promoting blood circulation. Based on the agreed thoughts of the Eastern and Western worlds, the application of Chinese herb and formulas for PBCRBS has valuable significance in the exploration of reducing the risk of cardiovascular event [10].

Blood-stasis syndrome has the status of platelet activation, and it has high correlation [11, 12]. As early as the last century of 1970s, there were scholars who had made pilot study to observe the mechanism of Chinese herb and formulas for PBCRBS on platelet function [13]. BSS has the definite diagnostic criteria [14] from 1991 in China, and during the past 5 years, diagnosis criteria have improved by scholars [15] and keep pace with the development of TCM. There is a special focus on natural compounds present in dietary and medicinal plants exhibiting antiplatelet/thrombotic properties. Now we know that platelet mainly was regulated by three kinds of substance, one kind is generated out of platelet such as catecholamine, collagen, thrombin, and prostacyclin; the second kind is generated from platelet and acts on the platelet membrane glycoproteins such as ADP, PGD_2_, PGE_2_, and 5-HT; the last kind is generated from platelet and acts on the platelet such as TXA_2_, cAMP, cGMP, and Ca^2+^, et al. Some of these substances have been identified as effective target of antiplatelet. Owing to the many problems of effectiveness and security of current antiplatelet drugs, a great need now arises to develop both efficacious and pharmaceutical medicines to combat these diseases. Screening the highly efficiency and fewer adverse effects of antiplatelet drugs from Chinese herb and formulas for PBCRBS attracts great attention of researchers, and the study of target or mechanism of Chinese herb and formulas for PBCRBS to be the hot topic of research and development of antiplatelet drugs. It had been approved that antiplatelet mechanism of Chinese herb and formulas for PBCRBS involves the following aspects.

## 2. Antiplatelet Mechanism of Chinese Herb and Formulas of Promoting Blood Circulation and Removing Blood Stasis

### 2.1. Inhibition of Platelet Aggregation

Platelet aggregation means the clumping together of platelets in the blood, which is the main function of platelet and has key role in the physiological hemostasia and pathogenesis of atherothrombosis. Platelet activates when it adheres to breakage of vessel or has been induced by activator. Activated platelet membrane glycoprotein (GP) IIb/IIIa exposes its fibrinogen receptor with the participation of Ca^2+^, one fibrinogen can bind to at least two GP IIb/IIIa at the same time, and platelet clump together with fibrinogen by GP IIb/IIIa. The typical aggregation is induced by different activators, which included the following two aspects, one is chemical agents such as ADP, collagen, thrombin, AA, and PAF, et al.; the other is shear stress. It is now taken that platelet aggregation rate (PAR) is the evaluation criterion of the intensity. Born [16] designed the platelet aggregation analyzer in 1962 by the turbidimetry principle which to accelerate the understanding of platelet aggregation. Now PAR was considered as the marker of antiplatelet efficacy evaluation and was used intensively in medical research of platelet. Studies show that the vast majority of Chinese herb and formulas for PBCRBS such as *Xiongshao Capsule* [17], *Compound Danshen dripping pills* [18], *Buyanghuanwu Decoction* [19], *Xuesaitong Capsule* [20], *Da Huang Zhe Chong pill* [21], and *Tongxingluo Capsule* [22], et al. can reduce the PAR of patients or animal model of thromboembolic diseases significantly. Active principles such as *ferulic acid* [23],* ligustrazine* [24], *propyl gallate* [25], *resveratrol* [26], *curdione* [27], *Total flavone in Sanguis Draconis *[28], *Salvianolic acid B* [29], *Hirulog* [30], and *Safflower flavin* [31] et al. can inhibit the platelet aggregation induced by AA, ADP, PAF, collagen, and thrombin to some extent, bringing out the superior antiplatelet effect.

### 2.2. Inhibition of Platelet Release Reaction

Platelet release reaction means that many substances stored in *α*-granules, dense granule, and lysosome in platelet are released out of platelet upon different activator. These substances including CD62p (P-selection), GPIIb/IIIa compound, PKC, *β*-TG, PF-4, and Ca^2+^, which has been considered as the usual evaluation indicator of screening the effective antiplatelet drug from Chinese herb and formulas for PBCRBS.

#### 2.2.1. CD62P

CD62p (P-selection) is a 140 kD glycoprotein which is present in the granules of platelets and translocates rapidly to the cell surface after platelet activation and is generally considered to be the gold marker of platelet activation [32, 33]. Clinical research indicates that the expression of CD62p increases markedly in the different types of cardiovascular patients (including patients with stable angina and ACS) [34–36] and has found high positive correlation between CD62p level and blood stasis syndrome (BSS) [37]. So making the increased expression of CD62p after platelet activation dropped is taken for the one of the antiplatelet mechanisms and scientific measurements of Chinese herb and formulas for PBCRBS. According to the current studies, *Danhong injection* [38],* Ligustrazine injection* [39], *Compound Danshen dripping pills* [40], *Taohongsiwu Decoction* [41], and *Tongxinluo capsule* [42] can reduce the CD62p expression after platelet activation significantly and inhibit platelet activation *in vivo*, to show satisfactory effect of antiplatelet.

#### 2.2.2. GPII b/III a Compound

The detection of PAC-1 is considered as the sensitive and important marker of platelet activation [43], PAC-1 is the specific monoclonal IgMK, which only binds to activating platelet GPIIb/IIIa compound, while it has no recognition capability for resting one. The activation of GPIIb/IIIa depends on the platelet activation which makes the former change its configuration to have strong affinity with receptors. Using the flow cytometry to detect PAC-1 which has the characteristic of specific fast sensitive, and has splendid future in the study on screening antiplatelet drugs from Chinese herb and formulas for PBCRBS.

*Da Huang Zhe Chong pill* is the earliest formula of PBCRBS and is widely used for atherothrombotic disease treatment. Research shows that it has better antiplatelet aggregation ability than aspirin [44], the further study indicates that it can reduce the level of PAC-1 after ADP-induced platelet activation and of patients with coronary heart disease and cerebral infarction in clinic, which also has superior antiplatelet activation than aspirin [21] and is an ideal antithrombotic drug. Other study [45] found that *Xue Fu Zhu Yu decoction* can inhibit the ADP-induced expression of GPIIb/IIIa compound significantly and restrain the ADP-induced platelet activation, which provides experimental evidence to long-term treatment of coronary heart disease, and no symptoms of myocardial ischemia, et al.

#### 2.2.3. PKC

protein kinase C (PKC), a ubiquitous protein kinase found in a variety of animal tissues, has been implicated in the regulation of many cellular processes and plays a central role in signal transduction. In platelets, the PKC is an important signaling mediator required for activation, secretion of granule contents, and aggregation [46]. During the process of platelet activation, close relationship between translocation of PKC in platelet and platelet function has been found. PKC has both cytosolic and plasma membranebound forms, and the former is the most abundant under resting conditions. The cytosolic form can translocate to the plasma membrane upon cell stimulation and elevation of cellular Ca^2+^, one particular and important aspect of PKC activation is the intracellular redistribution of the enzyme from the cytosol to the cell membrane [44]. Now translocation or redistribution of PKC from the cytosolic form to the plasma membrane can be taken for an indicator of PKC activation [47].

Resveratrol (*RESV*), a well-known polyphenolic compound of, was extracted from *Polygonum Cuspidatum*, which was a Chinese herb for PBCRBS and has been efficaciously used in traditional Chinese medicine to treat several diseases, including thromboembolic diseases for over hundreds of years. In recent years, pharmacological studies have found that *RESV *possesses multifaceted cardiovascular benefits, but the mechanism is not clear. Recent research [48] shows that PKC distributed mostly across the cytosol of platelets in resting platelets and redistributed to the membrane later to be activated by ADP. If pretreated by *RESV*, PKC translocation to the membrane was partially inhibited in the platelets activated by ADP. These results suggested that *RESV* inhibited the PKC-mediated signal transduction pathway in platelets, and it might act as an inhibitor on PKC activity in platelets and serve as a novel antithrombotic agent.

#### 2.2.4. PF-4 and *β*-TG

It is thought that PF-4 and *β*-TG are the specific indicators of platelet release reaction [49]. Both increases of PF-4 and *β*-TG indicate the height of platelet release reaction, which is common in thromboembolic disease and prethrombotic state. On the contrary, both decreases of PF-4 and *β*-TG indicate the suppression of platelet release reaction. *β*-TG can make the PGI_2_ concentration and adenylate cyclase activity reduction, and then make cAMP decrease which bring about weak inhibition and enhance the aggregation of platelet [50, 51]. PF-4 can reduce the anticoagulation of heparan sulphate in endothelial cell and enhance the metabolism of membrane phospholipid and AA, to produce TXA_2_, also PF-4 can promote precipitation and polymerization of fibrin monomer and accelerate platelet aggregation [52]. Research has found that *Salvia miltiorrhiza Bunge* injection [53] can reduce the PF-4 and *β*-TG concentration markedly to inhibit platelet aggregation.

#### 2.2.5. Ca^2+^

Calcium ion plays a vital role in the development of platelet activation. The transformation, aggregation, and release reaction of platelet are triggered by the increase of free calcium ion concentration of platelet ([Ca^2+^]_i_), which is the essential mechanism of thrombosis [54]. Studies have found that the increase of [Ca^2+^]_i_ in patient with CHD, meanwhile calcium antagonist can reduce [Ca^2+^]_i_ of platelet accompanied by inhibiting platelet aggregation [55].

**Figure 1:**
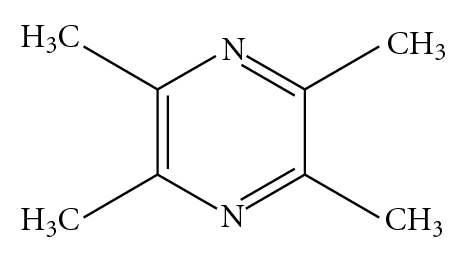
Chemical structures of ligustrazine.

**Figure 2:**
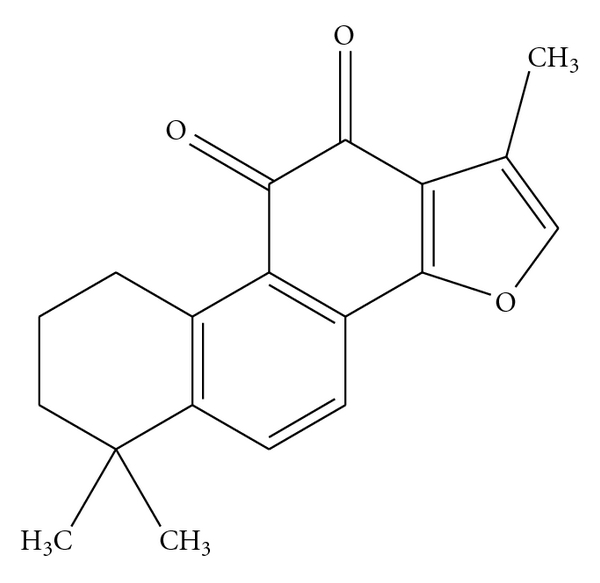
Chemical structures of Tanshinone IIA.

Studies [56] have indicated that some Chinese herbs, such as *Salvia Miltiorrhiza, Ligusticum wallichii Franch, Carthamus tinctorius, Radix Paeoniae Rubra, *and some active constituents as *Ligustrazine *(see Figure 1), Tanshinone IIA (see Figure 2), et al. have the certain effect of calcium channel antagonists and have good results of inhibit platelet aggregation and activation. Another research [57] shows that *Safflor yellow *(a kind of soluble natural pigment of *Carthamus tinctorius*) can inhibit platelet release of 5-HT and Ca^2+^, which has similar effect to *Ginkgolides* (admitted PAF receptor antagonist), which means that *Safflor yellow *might suppress the platelet activation via inhibition of PAF and calcium influx.

### 2.3. Influence of the Process of Platelet Metabolism

#### 2.3.1. Influence of the Metabolic System of Arachidonic Acid (AA)

TXA_2_ and PGI_2_ are the metabolites of AA, which have the strong bioactivity of PG, and have a short half-life, quickly degrad to the TXB_2_ and 6-keto-PGF1*α*, the latter make further metabolizes to the 6-keto-PGE. 

It is now thought that many cardiovascular diseases such as atherosclerosis, thrombosis, coronary spasm, acute myocardial infarction, and hypertension have close relationship with the disequilibrium of TXA_2_/PGI_2_ [58]. TXA_2_, which is synthesized and released by platelet microsome and has the function of promoting platelet aggregation and thrombosis, is one of the strong inducers of platelet aggregation and vasoconstrictor. TXA_2_ promotes the Ca^2+^ of density tube system free to make dense bodies contracting and releasing ADP and 5-HT, which result in platelet aggregation. PGI_2_ is the main metabolite of AA and is the strong endogenous inhibitor of platelet aggregation; it has the function of antiplatelet aggregation and vasorelaxant and is considered as the vascular protection factor. Under normal physiological state, TXA_2_ and PGI_2_ have the balance condition and keep the platelet internal environment stable. Out of balance of TXA_2_ and PGI_2_ in plasma or tissue is one of the reasons of platelet aggregation, vasospasm, and thrombosis. Studies show that influence of TXA_2_/PGI_2_ has been closely related to antiplatelet mechanism of Chinese herb and formulas for PBCRBS, such as *Total saponins of paeonia *[59] can reduce the ADP-induced platelet maximum aggregation rate and plasma TXB_2_ concentration, meanwhile, increase the plasma 6-keto-PGF1*α* concentration, which means it can promote the release of PGI_2_, inhibit the produce of TXA_2_, improve the balance of TXA_2_/PGI_2_, and reach the aim of antithrombotic therapy. The same results have been found in the following drugs: *Guanxin II* [60],* Taohongsiwu Decoction *[61],* Notoginsenoside *[62], *salvianolic acid A *[63], *Honghua injection *[64], et al.

#### 2.3.2. Influence of the Metabolic System of cAMP and cGMP

cAMP and cGMP in platelet are the second messengers of signal transmission, which make the different platelet activators acting on the specific receptor, then resulting in platelet aggregation and activation. Studies [65, 66] show that drugs which make the level of cAMP and cGMP increase can inhibit platelet aggregation owing to promoting the intake of calcium ions, lowering the level of Ca^2+^, and having close relation with the phosphorylation of myglobulin. So whether can affect the metabolic system of cAMP and cGMP has been taken as the main point of antiplatelet mechanism of Chinese herb and formulas for PBCRBS. Research [19] shows that *BuYangHuanWu decoction *can inhibit the ADP-induced platelet aggregation and the decrease of cAMP and cGMP after the platelet aggregation, which suggested that its antiplatelet aggregation may be related to inhibiting the decrease of cyclic nucleotide in platelets after the aggregation. *Compound Danshen dripping pills* [67] and *pseudoginseng* [68] have the same mechanism of antiplatelet.

### 2.4. Influence of the Signal Transduction in Platelet

There is a series of signal transductions in platelet, which has close relationship with platelet activation. Upon agonist stimulation, specific receptor of membrane binds to the ligand to make the conformational changes and to activate the key enzymes action, which produces or releases the signal molecules and led to adhesion, aggregation, and reaction release to form thrombus at last. The mechanism of transmembrane signal transduction in platelet is unclear owing to more than one receptor bound by platelet agonist and the activated platelet release *α*-granules as secondary agonist to bring about amplification effect [69]. Platelet signal transduction pathway usually includes several aspects [70]: PI3-K pathway, PLC-*β* pathway, PTK pathway, MARK pathway, cAMP-PKA pathway, and PLA_2_ pathway. At present, most researches are about Phosphoinositide 3-kinase (PI3K). PI3K is a critical transmitter of intracellular signaling during platelet activation. The PI3K family is divided into three classes (I, II, and III). Depending on differences in the heterodimerization of catalytic subunits and regulatory subunits, class I is further divided into IA (PI3K*α*, PI3K*β*, and PI3K*γ*) and IB (PI3K*δ*), PI3K*β* and PI3K*γ* are crucial in platelet signaling [71]. Akt phosphorylation can be used as an indicator of PI3K pathway activation [72, 73].

**Figure 3:**
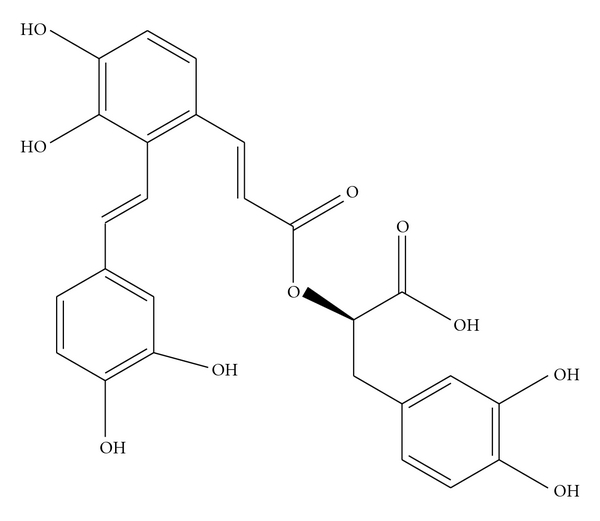
Chemical structures of salvianolic acid A.

In recent years, with the further study of antiplatelet mechanism of Chinese herb and formulas for PBCRBS, there are studies involving signal transduction in platelet to investigate the mechanism. *Salvianolic acid A* (SAA, Figure 3) is a water-soluble component from the root of *Salvia miltiorrhiza Bunge*, a herb that is widely used for atherothrombotic disease treatment in China. New study [74] shows that SAA could inhibit platelet spreading on fibrinogen, a process mediated by outside-in signaling. Western blot analysis showed that SAA, like the PI3K inhibitors LY294002 and TGX-221, potently inhibited PI3K, as shown by reduced akt phosphorylation, which indicates that the target spot may be the PI3K*β*. The *in vitro* findings were further evaluated in the mouse model of arterial thrombosis, in which SAA prolonged the mesenteric arterial occlusion time in wild-type mice. Interestingly, SAA could even counteract the shortened arterial occlusion time in Ldlr^tmlHer^ mutant mice. And for the first defined the fact [74] that SAA inhibits platelet activation via the inhibition of PI3K and attenuates arterial thrombus formation *in vivo*. The results suggest that SAA may be developed as a novel therapeutic agent for the prevention of thrombotic disorders. 

## 3. Discussion and Perspective

 From above mentioned, during the past 30 years, research of antiplatelet and antithrombotic therapy of Chinese herb and formulas for PBCRBS has made rapid progress, but there are still some problems existing. In the clinical research, at present many studies limited to small sample of curative effects, lack of multicenter, prospective, large sample, and control study which made the clinical practice of Chinese herb and formulas for PBCRBS be short of definite clinical evidence. And Chinese scholars has begun to attempt to study like above and got to some good results [75]. But those which deserve attention are, in the practical use of clinical medicine, we should comply with the principle of differentiation of symptoms and signs, minimize the potential abuse, and improve on the clinical practical effects. In the experimental research, many studies mainly focused on the mechanism on one aspect of a certain Chinese herb and formulas for PBCRBS, the experimental design owes rigor, and only a few studies were equipped with *in vitro* and *in vivo* at the same design. It is generally known that platelet activation is a complex, multifactor process, which involves adhesion, aggregation, and reaction release, for example, there are different platelet activation stimulators, which have the different mechanism of platelet aggregation and signal transduction, it is necessary to take a systematic study on the mechanism of Chinese herb and formulas for PBCRBS inhibiting platelet aggregation by different stimulators in the future and making further study on the signal transduction in platelet, now Chinese scholars [74] have made good study and publish the paper on the well-famous journal.

 Proteomics technology has been successfully applied to platelet research, contributing to the emerging field of platelet proteomics which led to the identification of a considerable amount of novel platelet proteins, many of which have been further studied at functional level [76]. During the last 3 years, a rapid development of two-dimensional gel electrophoresis and mass spectrometry-based proteomic approaches has been used to profile alterations in platelet proteins [77–79]. Using differential proteomics of platelet, our previous studies found many different platelet proteins [37, 80] between CHD patients of blood stasis syndrome (BSS) and non-BSS patients, and healthy controls, which indicate that the platelet cytoskeleton may play an important role in the development in BSS of CHD. Based on the Chinese medicine principle of “prescription and syndrome are corresponding”, these platelet differential proteins may be the new target spots or target group. Getting intensive study on it, we believe that we can develop many new antiplatelet and antithrombolytic drugs possess definite curative effect and target, clear mechanism.

## Figures and Tables

**Table 1:** The ingredient of frequently used formulas for promoting blood circulation and removing blood stasis.

Names of formulas	Ingredients of formulas	Label
Xiongshao capsule	*Szechuan Lovage Rhizome, Red Paeony Root*	Chinese patent drug
Compound danshen dripping pills	*The root of red-rooted salvia, Panax notoginseng, Borneol*	Chinese patent drug
Buyanghuanwu decoction	*Radix Astragali Bunge, Peach Seed, Safflower, Szechuan Lovage Rhizome, Angelica sinensis, Red Paeony Root, earthworm*	
Xuesaitong capsule	*Panax Notoginsenosides*	Chinese patent drug
Tongxinluo capsule	*Sanguisuge, Scorpio, centipede, ground beeltle, cicada slough,* et al.	Chinese patent drug
Danhong injection	*The root of red-rooted salvia, safflower*	Chinese patent drug
Taohongsiwu decoction	*Peach Seed, Safflower, Szechuan Lovage Rhizome, Angelica sinensis, white paeony root, Radix Rehmanniae Praeparata*	
Xue Fu Zhu Yu decoction	*hovenia dulcis, radix achyranthis bidentatae, peach seed, safflower, Szechuan Lovage Rhizome, Angelica sinensis, white paeony root, Radix Rehmanniae Praeparata, radix bupleuri, Platycodon grandiflorum,* et al.	
